# Response of Yields, Soil Physiochemical Characteristics, and the Rhizosphere Microbiome to the Occurrence of Root Rot Caused by *Fusarium solani* in *Ligusticum chuanxiong* Hort.

**DOI:** 10.3390/microorganisms12112350

**Published:** 2024-11-18

**Authors:** Xiaofang Sun, Yong Liu, Lian He, Zaiyin Kuang, Shundong Dai, Lixia Hua, Qiuping Jiang, Taiyang Wei, Pengsheng Ye, Hualan Zeng

**Affiliations:** Industrial Crops Research Institute, Sichuan Academy of Agricultural Sciences, Chengdu 610300, China; sunxiaofang207@163.com (X.S.); liuyong918@126.com (Y.L.); yz321411@126.com (L.H.); kuangzaiyin@scsaas.cn (Z.K.); dai_shun_dong@126.com (S.D.); pagenew@163.com (L.H.); jqp19852024@126.com (Q.J.); weity1991@163.com (T.W.); yeps18@163.com (P.Y.)

**Keywords:** rhizosphere microbiome, *Ligusticum chuanxiong*, root rot, soil physicochemical properties, biological control

## Abstract

*Ligusticum chuanxiong* Hort. is considered an important medicinal herb with extremely high economic value and medicinal value due to its various effects, including anti-oxidation, sedative action, hepatoprotection, and invigorating blood circulation. However, *L. chuanxiong* cultivation is hampered by various plant diseases, especially the root rot caused by *Fusarium solani*, hindering the sustainable development of the *L. chuanxiong* industry. The occurrence of soil-borne diseases is closely linked to imbalances in the microbial community structure. Here, we studied the yields, rhizosphere microbiota, and soil physiochemical characteristics of healthy and diseased *L. chuanxiong* plants affected by root rot with high-throughput sequencing and microbial network analysis, aiming to explore the relationships between soil environmental factors, microbiomes, and plant health of *L. chuanxiong*. According to the results, *L. chuanxiong* root rot significantly decreased the yields, altered microbial community diversity and composition, enriched more pathogenic fungi, recruited some beneficial bacteria, and reduced microbial interaction network stability. The Mantel test showed that soil organic matter and pH were the major environmental factors modulating plant microbiome assembly. The root rot severity was significantly affected by soil physiochemical properties, including organic matter, cation exchange capacity, available nitrogen, phosphorus, potassium, and pH. Furthermore, two differential microbes that have great potential in the biocontrol of *L. chuanxiong* root rot were dug out in the obtained results, which were the genera *Trichoderma* and *Bacillus*. This study provided a theoretical basis for further studies revealing the microecological mechanism of *L. chuanxiong* root rot and the ecological prevention and control of *L. chuanxiong* root rot from a microbial ecology perspective.

## 1. Introduction

*Ligusticum chuanxiong* Hort., a prominent and extensively utilized form of traditional Chinese medicine, belongs to the Umbelliferae family. This perennial herb is primarily cultivated in Sichuan Province, China, where it contributes to over 90% of the national yield [1]. The desiccated rhizome and extracts of *L. chuanxiong* have potential applications in the treatment of headaches as well as cardiovascular and cerebrovascular disorders [2]. *L. chuanxiong* has been shown to possess sedative, analgesic, antispasmodic, and antibacterial properties, making it effective in the treatment of conditions such as migraine, rheumatic arthralgia, and cardiovascular diseases. Contemporary pharmacological research indicates that it enhances renal blood flow, inhibits lipid peroxidation, and mitigates pulmonary fibrosis. In addition to its medicinal uses, *L. chuanxiong* serves as a significant raw material in the formulation of health and skincare products, including *L. chuanxiong* mutton soup and fish head soup. In 2020, the annual production of *L. chuanxiong* rhizome reached 20,000 metric tons, with an estimated value exceeding USD 155 million [1]. Consequently, *L. chuanxiong* possesses significant economic and medicinal importance.

In recent years, the incidence rate of *L. chuanxiong* has risen to approximately 50% to 60% during harvest seasons in Pengzhou and Dujiangyan, and in severe instances, it can reach as high as 80% [3]. This increase can be attributed to a prolonged absence of effective control measures, which has a direct negative impact on both the yield and quality of medicinal materials, resulting in significant economic losses in the production of *L. chuanxiong*. At present, a variety of chemical and agricultural strategies have been implemented to prevent and manage root rot. While these measures have acquired some degree of effectiveness, they remain inadequate for comprehensive disease control [3]. Additionally, prolonged and widespread application of chemical fungicides is likely to enhance the resistance of pathogens to these agents, leading to issues such as pesticide residues and environmental contamination. Consequently, there is an imperative requirement to address this detrimental disease through the development of sustainable, effective, and environmentally responsible control strategies [4].

A multitude of studies have indicated that an imbalance in rhizospheric microbial diversity is associated with the emergence of soil-borne diseases [5,6,7,8,9]. Consequently, the health of soil and the diversity of beneficial microorganisms are considered essential determinants for the promotion of robust crop production and the management of diseases [10,11]. An understanding of the composition of the rhizosphere microbial community and its response to environmental factors is essential for the effective management of root rot. While several studies have addressed the management of root rot in *L. chuanxiong*, there remains a paucity of in-depth research concerning the diversity of rhizosphere microorganisms in response to *F. solani* infection. Additionally, critical factors influencing the progression of root rot and the potential mechanisms for its inhibition have yet to be thoroughly investigated.

This study aimed to uncover the key environmental factors and core microbes influencing the incidence of *L. chuanxiong* root rot, as well as the microbial populations that proliferate as a result of pathogen infection in the plant. Additionally, the study sought to examine the relationships among the pathogen, soil environmental variables, and the rhizosphere microbial community, with the aim of elucidating the microbial ecological mechanisms underlying the occurrence of *L. chuanxiong* root rot. This study examined the variations in soil physicochemical properties and the microbial communities present in the rhizosphere of healthy and diseased *L. chuanxiong*. The distinct characteristics of the soil associated with both healthy and diseased plants were elucidated. Furthermore, the microbes isolated and identified from the rhizosphere soil may serve as promising beneficial microorganisms for biocontrol applications, thereby facilitating future research and development endeavors.

## 2. Materials and Methods

### 2.1. Field Experiment and Sample Collection

The experiment was carried out across fields matching the following conditions: (1) the presence of *L. chuanxiong* for more than 10 years; (2) a high incidence of more than 45% severe infection with root rot in soil; (3) the distance between the sampling sites was close (less than 5 km in a straight line). According to the above conditions, six fields located in six villages within Aoping Town, Pengzhou City, Sichuan Province (104.0214° E, 31.0811° N) were selected as the sampling sites. All fields were subjected to uniform cultivation and agricultural practices. *L. chuanxiong* seedlings, sourced from the Tongyi Farmers’ Professional Cooperation for Crop Seed Sales in Pengzhou, were planted on 20 September 2022. The planting configuration consisted of a row spacing of 20 cm, with individual plants spaced 15 cm apart.

The selected pathogen was *Fusarium solani* (SAAS-2204), which was obtained from Sichuan Academy of Agricultural Sciences (SAAS). The inoculum preparation was first performed by incubating *F. solani* on PDA medium at 26 °C until it sporulated. Then, spore collection was performed and was diluted to obtain the final application *F. solani* spore suspension (1 × 10^6^ spores mL^−1^). Finally, 10 mL of spore suspension per plant was inoculated to the root of *L. chuanxiong* by irrigation 20 days after emergence. *L. chuanxiong* plants at the rhizoma swelling stage that showed no diseased spots on their rhizoma or brown withered stems were defined as healthy; plants with diseased spots on their rhizoma and hollow stems with brown xylem were classified as diseased. The sampling of all plants involved in this experiment occurred between 20 April and 22 May 2023. In each field of 20 m × 20 m, five sampling points were selected, with 10 healthy and 10 diseased *L. chuanxiong* specimens randomly selected. And their roots were carefully dug up. The shoots and rhizomes were subsequently separated to determine their fresh weight. Fifty healthy or diseased *L. chuanxiong* plants’ rhizosphere soil from the same field was mixed as one replicate, resulting in six replicates in both healthy and diseased plants at all sampling sites. Following this, the samples were transported to the laboratory, where they were dried separately in an oven at 70 °C for 72 h, after which their dry weight was recorded. In addition, the rhizosphere soil samples were meticulously collected. The excess soil was manually shaken from the roots, leaving approximately 1mm of soil remained attached to the roots. Subsequently, the rhizosphere soil was obtained from the 1 mm layer of soil using a sterile brush and transferred directly into 1.5 mL centrifuge tubes. The soil samples were then stored in an ice box and transported to the laboratory within 12 h. The soil samples were split into two sections: one was kept at −80 °C for total DNA extraction, while the other was subjected to physicochemical analyses following air drying. The severity of *L. chuanxiong* root rot was assessed using the grading criteria established by Li [12]. Subsequently, the disease index was calculated based on these evaluation results. The calculation formula used is as follows:

Disease index = (Σ (number of affected plants at each severity level × corresponding relative value))/(total number of plants under examined × maximum value of disease scale) × 100

### 2.2. Physicochemical Properties of Rhizosphere Soil

The methods described in previous studies were adopted for the soil physicochemical properties determination. Soil pH was measured using a pH meter (Sartorius, Shenzhen, China) in a 1:2.5 soil-to-water 1 M KCl solution according to the agricultural industry standard of the People’s Republic of China (NY/T 1377-2007 [13]). The cation exchange capacity (CEC) was assessed utilizing the ammonium saturation diffusion technique. Organic matter (OM) was measured by the K_2_CrO_7_-H_2_SO_4_ oxidation method [14]. The semi-micro Kjeldahl digestion technique was utilized to evaluate the total nitrogen (TN) content in soil, as reported by Fei et al. [15]. Similarly, the total phosphorus (TP) concentration was analyzed by molybdenum antimony colorimetry after using the HClO_4_-H_2_SO_4_ digestion method [16]. The NaOH fusion-flame atomic absorption spectrometry technique was used to determine total potassium (TK) content. Available nitrogen (AN) was quantified using the alkaline permanganate method [17]. The extraction of soil available phosphorus (AP) was conducted using a 0.5 M sodium bicarbonate (NaHCO_3_) solution at a pH of 8.5, followed by analysis through the molybdenum antimony anticolorimetric method, employing an ultraviolet–visible spectrophotometer (UV-2600; Mettler-Toledo, Shanghai, China) at a wavelength of 700 nm, as outlined by Feng et al. [18]. Soil available potassium (AK) concentrations were determined using the ammonium acetate (NH_4_OAc) extraction method [19].

### 2.3. DNA Extraction and Sequencing

Total DNA was extracted from 250 mg of rhizosphere soil utilizing the Powersoil DNeasy DNA isolation kit (QIAGEN Laboratories, Hilden, Germany), following the manufacturer’s guidelines. Subsequently, the quality and concentration of the extracted DNA were assessed using 1% agarose gel electrophoresis and a NanoDrop^®^ ND-2000 Spectrophotometer (Thermo Scientific^®^, Wilmington, DE, USA). In addition, the QUBIT^®^ 2.0 fluorometer (Life Technologies, Carlsbad, CA, USA) was employed to quantify the DNA concentration (ng·µL^−1^), with all samples adjusted to a final volume of 20 µL at a concentration of 5 ng·µL^−1^. Subsequently, the samples were lyophilized and dispatched to Novogene Co., Ltd. (Beijing, China) for sequencing of the 16S rRNA and ITS regions.

The resulting amplicons for both the 16S rRNA gene and the ITS1 region were sequenced using the Illumina NovaSeq platform (Novogene, Beijing, China) in accordance with established protocols. For bacterial community profiling, R programming language (version 4.10) was used for the bioinformatics pipeline and subsequent analyses. The cutadapt plugin version 2.10 (DADA2 package) was used to remove the forward and reverse primers from the NovaSeq sequencing reads. Data processing and taxonomic assignment were performed through the DADA2 pipeline.

### 2.4. Statistical Analysis

An analysis of variance (ANOVA) was conducted using SPSS version 19.0 (Suzhou C&J Marketing Software Co., Ltd.) to assess statistical significance. The Duncan multiple range test was employed to identify significant differences between the healthy and diseased groups, with a *p*-value of less than 0.05 considered indicative of statistical significance. Other statistical analyses were conducted utilizing R version 4.2.1 (Revolution Analytics, Microsoft). Linear regression analysis (Pearson correlation) was conducted to ascertain the relationships between the disease index and certain keystones or specific microbial taxa. Additionally, the linear discriminant analysis effect size (LEfSe) methodology was employed to statistically assess the differential abundance of OTUs. Furthermore, Mantel tests were performed to determine the correlations between soil properties and microbial communities utilizing the ‘vegan’ package in R. The interrelationships among various soil properties were evaluated through Pearson correlation analysis and represented visually through a heatmap. Statistical significance was established at a threshold of *p* < 0.05.

The co-occurrence network was developed in accordance with methodologies outlined in prior research by assessing the correlations among genera within both healthy and diseased cohorts, utilizing the “Hmisc” package in R version 4.2.1 [20]. Co-occurrence was deemed stable when the Spearman correlation coefficient was greater than 0.8 or less than −0.7, with a *p*-value of less than 0.05, following adjustment using the Benjamini–Hochberg procedure. The topological characteristics of the network were assessed in accordance with previously established methodologies [21].

## 3. Results

### 3.1. Effect of Root Rot Disease on the Yield of Ligusticum chuanxiong

**Figure 1:**
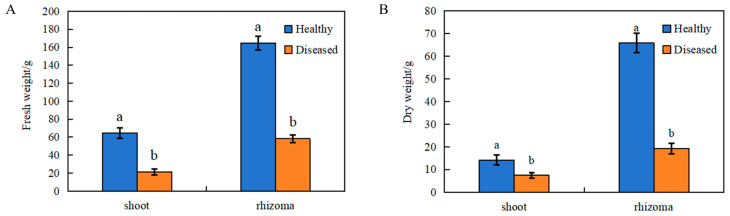
Effects of root rot on the fresh weight (**A**) and dry weight (**B**) of both the shoot and rhizoma in *L. chuanxiong*. Different lowercase letters positioned above the error bars indicate statistically significant differences (*p* < 0.05; Duncan’s test).

Significant differences in both the fresh weight and dry weight of *L. chuanxiong* were detected following exposure to pathogen infection. The occurrence of root rot markedly impeded the growth of the plants. In the context of the study, the fresh weight and dry weight of shoots exhibited reductions of 67.23% and 47.89%, respectively. Meanwhile, the fresh weight and dry weight of rhizomes demonstrated decreases of 64.68% and 70.75%, respectively (Figure 1). Furthermore, regression equations were developed to analyze the correlation between the fresh weight and dry weight of *L. chuanxiong* and the root rot disease index. The findings indicated that both the fresh weight and dry weight of the rhizoma demonstrated heightened sensitivity in response to elevated pathogen loads (Table 1). The cultivation of *L. chuanxiong* was primarily focused on the production of tuberous roots; consequently, the dry weight of the rhizoma was utilized as a metric to evaluate the growth of *L. chuanxiong* under pathogen-induced stress. Based on the above results, root rot significantly affected the growth and reduced the yield of *L. chuanxiong*, especially the rhizoma.

### 3.2. General Characteristics of Illumina NovaSeq PE250 Sequencing Results

**Figure 2:**
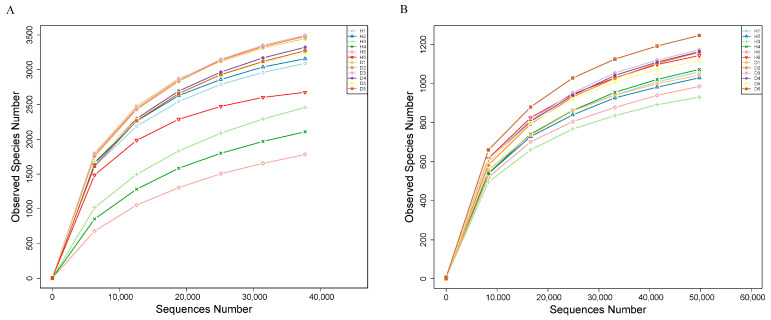
Rarefaction curves for rhizosphere soil bacteria (**A**) and fungi (**B**). The letter H indicates healthy plants, while D denotes diseased plants.

Based on the barcode-labeled sequence, a total of 12 soil samples were identified and differentiated. From these samples, a cumulative total of 1,008,575 circular consensus sequencing (CCS) reads, annotated with fungal species, was acquired. The number of CCS reads per sample varied between 79,046 and 90,873, yielding an average of 84,048 reads per sample. A cumulative total of 833,623 CCS reads, annotated with bacterial species, was acquired. The number of CCS reads for each sample varied, ranging from 57,627 to 86,595, with an average of 69,469 CCS reads per sample. Following the filtering of barcoded-CCS reads for quality and the elimination of chimeras, over 98% of the effective reads were achieved. Each sample yielded an average of 64,482 fungal optimized-CCS reads, with more than 95% of the reads maintaining a quality score ≥30. Each sample generated an average of 52,265 optimized bacterial CCS reads, with 97% effective sequences, and over 93% of the reads had a quality score of ≥30 (Table S1). Obviously, the rarefaction curves for bacterial and fungal OTUs began to level off once the sequencing quantity for both fungi and bacteria hit 20,000, which implies that the samples encompassed the majority of the microbiomes present in the rhizosphere (Figure 2). These results suggested that the data were adequate for further statistical analysis.

### 3.3. Venn Diagrams Analysis of OTUs

**Figure 3:**
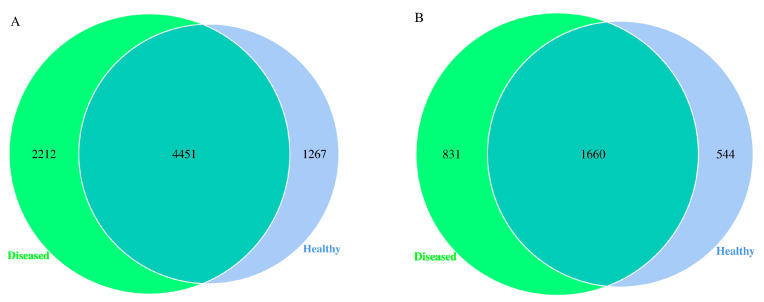
OTU Venn diagram analysis of rhizosphere soil bacteria (**A**) and fungi (**B**).

On average, 12 soil samples generated 7930 bacterial OTUs and 3035 fungal OTUs, achieving coverage rates of over 97% and 98%, respectively. This suggests that the sequencing depth for all samples was adequate, making it appropriate for further analysis of the microbial communities present in the samples. The Venn diagrams visually represented the overlap of OTUs between healthy and diseased samples, showing both shared and unique OTUs. The analysis of bacterial OTUs revealed that 4451 OTUs were common to both healthy and diseased samples, while there were 1267 unique to healthy samples and 2212 unique to diseased samples (Figure 3A). Fungal OTUs analysis indicated that 1660 OTUs were shared by healthy and diseased samples, while the number of fungal OTUs that were unique to healthy samples was 544, and for diseased samples, it was 831 (Figure 3B). When exposed to pathogen invasion, the quantity of bacterial and fungal OTUs unique to rhizosphere soil increased in comparison to that of healthy plants. Therefore, after the infection of root rot, new species of bacteria and fungi have appeared in rhizosphere of *L. chuanxiong*. From the perspective of species, convergence and variation coexist, and the community structure has undergone complex and subtle changes.

### 3.4. Response of Alpha Diversity to Healthy and Diseased Plants Rhizosphere Soil

**Figure 4:**
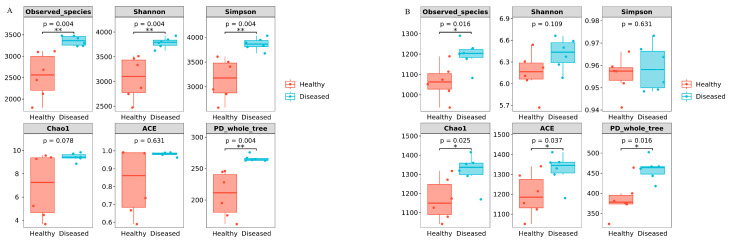
Alpha diversity indexes between the healthy and diseased plants rhizosphere soil bacteria (**A**) and fungi (**B**). * *p* < 0.05; ** *p* < 0.01.

Alpha diversity was used to evaluate the abundance, variety, and uniformity of bacteria and fungi present in the rhizosphere soil. In the case of bacteria, the diseased rhizosphere soil possessed notably higher values of Shannon, Simpson, and PD_whole tree indices compared to the healthy soil, indicating a greater bacterial diversity in the diseased soil (*p* < 0.05, Figure 4A). A minimal increase and difference were noted in Chao1 and ACE, suggesting that there is no significant variation in bacterial richness and evenness between healthy and diseased soil (*p* > 0.05, Figure 4A). In the case of fungi, the diseased soil exhibited higher levels across all metrics, with notable differences found in the Observed_species index as well as in the Chao1 and ACE indices (*p* < 0.05, Figure 4B). This indicates a comparatively greater fungal richness in the diseased soil. The variation in the Shannon and Simpson indices between healthy and diseased soil was not statistically significant (*p* > 0.05, Figure 4B), indicating that there is a similar level of diversity and evenness in both types of soil. Notably, the indexes for bacteria and fungi in diseased soil were considerably greater than those in healthy soil, indicating a higher abundance, diversity, and evenness of bacterial and fungal populations in the diseased soil compared to the healthy soil (Table S2).

### 3.5. Rhizosphere Bacterial and Fungal Community Assembly

**Figure 5:**
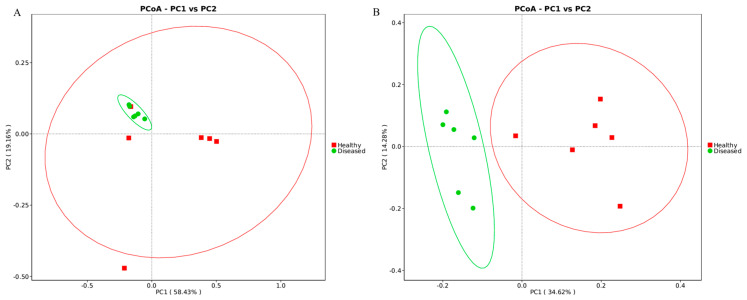
Principle coordinates analysis of dimension reduction analysis based on Bray−Curtis distances according to the abundance of rhizosphere bacteria (**A**) and fungi (**B**).

The results of the PCoA, utilizing Bray–Curtis distances, indicate that the composition of rhizosphere bacteria exhibited certain similarities between healthy and diseased soils (Figure 5A). In contrast, the rhizosphere fungal communities in these soils displayed significant differences and were distinctly separated (Figure 5B). Furthermore, the first two principal components (PC1 and PC2) of the PCoA collectively explained 48.90% of the variance observed in rhizosphere fungal communities.

**Figure 6:**
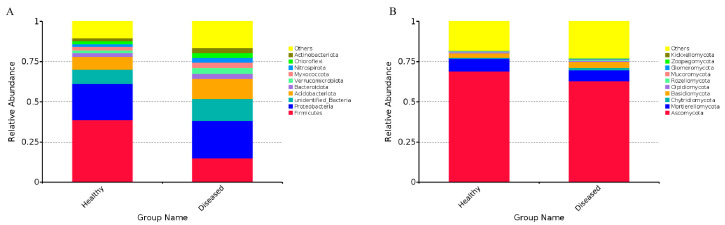
Taxonomic summary of the relative abundance of the rhizosphere bacterial phyla (**A**) and fungal phyla (**B**) in healthy and diseased soil.

The rhizosphere microbiota exhibited significant differences between healthy and diseased soils at both the phylum and genus levels. At the phylum level, the rhizosphere bacterial communities were predominantly composed of Firmicutes, Proteobacteria, unclassified taxa, Acidobacteriota, Bacteroidota, Verrucomicrobiota, Myxococcota, Nitrospirota, Chloroflexi, and Actinobacteriota, collectively representing 83.6% to 89.7% of the identified phyla (Table S3). Furthermore, a notable disparity was identified in the abundance of the Firmicutes, which exhibited a significantly higher proportion in healthy soil (*p* < 0.05, Figure 6A). In contrast, the distribution of other phyla was relatively comparable between healthy and diseased soil. Nevertheless, Ascomycota, Mortierellomycota, Chytridiomycota, and Basidiomycota represented a substantial portion of the fungal community, serving as the predominant phyla in both healthy and diseased soil (Figure 6B, Table S4). Moreover, Ascomycota and Mortierellomycota exhibited greater abundance in healthy soil compared to diseased soil, whereas the abundance of Chytridiomycota and Basidiomycota was found to be lower in healthy soil.

**Figure 7:**
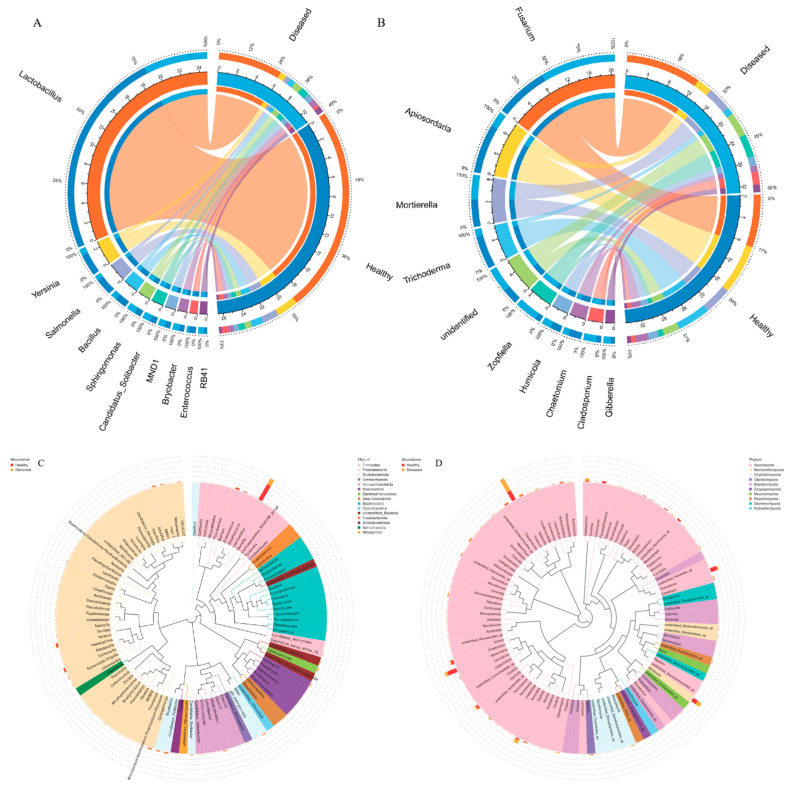
Taxonomic summary of the relative abundance of the rhizosphere bacterial genera (**A**) and fungal genera (**B**) in healthy and diseased soil; Phylogenetic trees of the top 100 genera of bacteria (**C**) and fungi (**D**) based on their relative abundance.

At the genus level, our analysis identified a total of 704 bacterial genera. Among these, seven genera exhibited a relative abundance of ≥1%, which were *Lactobacillus*, *Bacillus*, *Yersinia*, *Salmonella*, *MND1*, *Sphingomonas*, and *Candidatus Solibacter* (Table S5; Figure 7A). It is noteworthy that the highest prevalence of Lactobacillus was observed in healthy soil, accounting for 32.73%, which is significantly greater than its presence in diseased soil. Concurrently, the proportions of *Bacillus*, *Yersinia*, *Salmonella*, and *Enterococcus* were found to be higher in healthy soil. In contrast, diseased soil exhibited an increased abundance of *Sphingomonas*, *Candidatus Solibacter*, *MD1*, *RB41*, and *Bryobacter*. Additionally, a comprehensive analysis revealed the identification of 676 distinct fungal genera, among which 12 genera were identified as dominant, each contributing to a relative abundance of ≥1% (Table S6). The abundance of the genera *Trichoderma*, *Apiosordaria*, *Mortierella*, *Chaetomium*, and *Humicola* was found to be greater in healthy soil compared to diseased soil. Conversely, the genera *Fusarium*, *Unclassified*, *Zopfiella*, *Cladosporium*, and *Gibberella* exhibited an increased abundance in diseased soil (Figure 7C). The prevalence of *Trichoderma* was found to be markedly higher in healthy soil, exhibiting a relative abundance of 8.60%, in contrast to the 1.83% observed in diseased soil. This suggests that *Trichoderma* may be instrumental in the biological control of root rot in *L. chuanxiong*. Additionally, phylogenetic trees were constructed for the top 100 bacterial and fungal genera based on their abundance (Figure 7B,D), which illustrate the comparative abundance of these genera in both healthy and diseased soil.

According to the results, it can be seen that the microbial composition in the rhizosphere of *L. chuanxiong* showed complex and subtle changes after root rot infection. Some beneficial microorganisms, such as Firmicutes, Ascomycota, and Mortierellomycota, or *Bacillus* and *Trichoderma*, were recruited for resisting the invasion of pathogens.

### 3.6. Connection Between Microbial Communities and Environmental Factors

**Figure 8:**
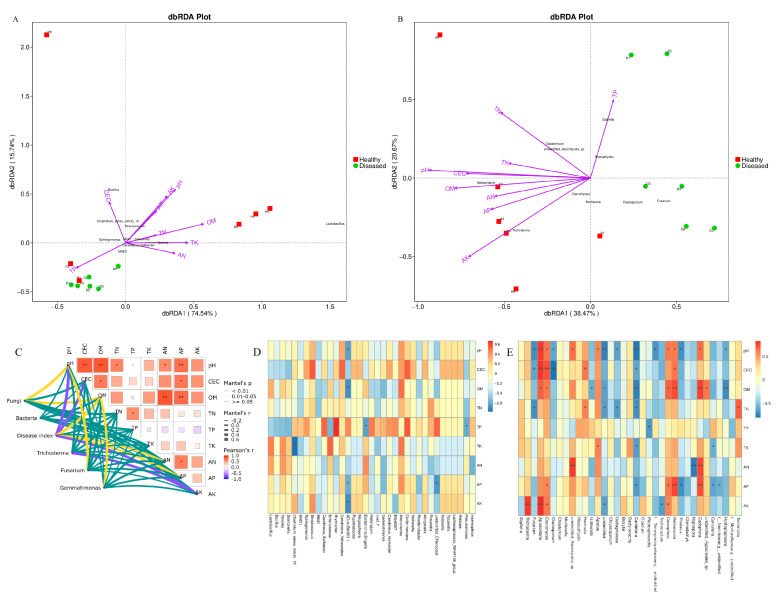
Connection between microbial communities and environmental factors. (**A**,**B**) Redundancy analysis triplots of bacterial (**A**) and fungal (**B**) communities; (**C**) The Mantel test, which indicates the correlation between microbial community and soil properties and their pairwise comparisons through pearson correlations; (**D**,**E**) Pearson correlation coefficient diagrams for the top 35 bacterial (**D**) and fungal (**E**) genera in relation to soil environmental factors. CEC: cation exchange capacity; OM: organic matter; TN: total nitrogen; TP: total phosphorus; TK: total potassium; AN: available nitrogen; AP: available phosphorus; AK: available potassium. * denotes significance *p* < 0.05. ** represent significance *p* < 0.01.

The association between multiple environmental factors and the composition of microbial communities was clarified through the use of redundancy analysis. The findings indicated that pH, OM, AK, and TP were significant determinants in shaping both bacterial and fungal communities (Figure 8A,B). Additionally, CEC and TN in the soil had a notable impact on the assembly of fungal communities (Figure 8B). In addition, the Mantel test revealed that there was no notable correlation between the rhizosphere bacteria and the soil environmental factors. In contrast, a significant positive correlation was found between pH and the concentration of soil OM with the rhizosphere fungi (*p* < 0.05, Figure 8C). Furthermore, the severity of root rot was notably influenced by pH, CEC, and the levels of soil OM, AN, AP, and AK (Figure 8C; Table S7). Correlation analysis conducted at the genus level indicated that the genus Lactobacillus, which exhibited the highest abundance in healthy soil, demonstrated a positive correlation with the concentrations of soil OM, AN, and TK (Figure 8D). Moreover, a highly significant positive correlation was identified between the concentration of soil AK and the abundance of Trichoderma and Apiosordaria (*p* < 0.01, Figure 8C,E). In contrast, the abundance of Fusarium exhibited a significant negative correlation with pH, CEC, and TN (*p* < 0.05, Figure 8C,E). Additionally, the concentration of soil OM demonstrated a substantial influence on Gemmatimonas (Figure 8C). These findings suggest that pH and OM may play a critical role in shaping the structure of the fungal community. Maybe it is helpful to control root rot by raising soil pH or increasing potassium fertilizer content.

**Figure 9:**
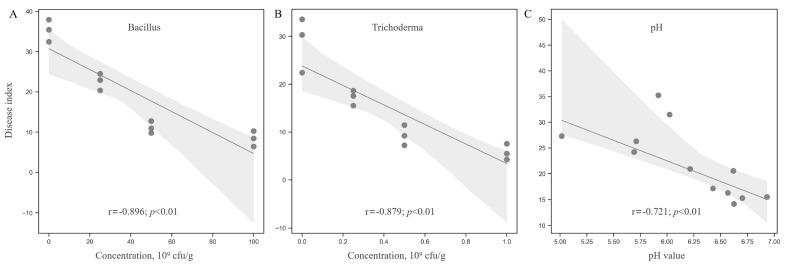
The interconnections among keystone microbiomes, soil pH levels, and the disease index associated with root rot. (**A**) Disease index of root rot in response to the concentration of *Bacillus*; (**B**) Disease index of root rot in response to the concentration of *Trichoderma*; (**C**) Disease index of root rot in response to soil pH. The shaded area represents the 95% confidence interval for the corresponding regression lines.

Interestingly, a noteworthy correlation was established between the severity of root rot and the levels of *Trichoderma* and *Bacillus*. To confirm the effect of keystone microbiome, certain strains of *Trichoderma* and *Bacillus* were isolated from the soil samples, and confrontation tests against *F. solani* were performed, demonstrating their ability to inhibit the growth of *F. solani*. The hyphae growth of the pathogen on potato dextrose agar (PDA) medium was significantly reduced with the introduction of *Trichoderma* (Figure S1) and *Bacillus* (Figure S2). Additionally, non-volatile organic compounds from *Trichoderma* were obtained, which demonstrated a notably strong ability to inhibit the growth of *F. solani* (Figure S3). Furthermore, the regression analysis revealed that an increase in *Bacillus* concentration was associated with a decrease in the root rot disease index (Figure 9A). A significant negative correlation was found between the disease index and the concentrations of both *Trichoderma* and *Bacillus*, suggesting that variations in microbial populations affect the severity of root rot (Figure 9B). Likewise, soil pH exhibited significant negative correlations with the severity of root rot as well as with the concentrations of *Trichoderma* and *Bacillus* (Figure 9C). The findings indicate that certain microbiomes present in healthy soil may play a crucial role in inhibiting the progression of root rot. Following an assessment of their colonization capabilities and biocontrol efficacy in field conditions, *Trichoderma* and *Bacillus* could be cultivated and utilized as promising biocontrol agents for the management of root rot.

### 3.7. Co-Occurrence Networks of Microbial Communities

**Figure 10:**
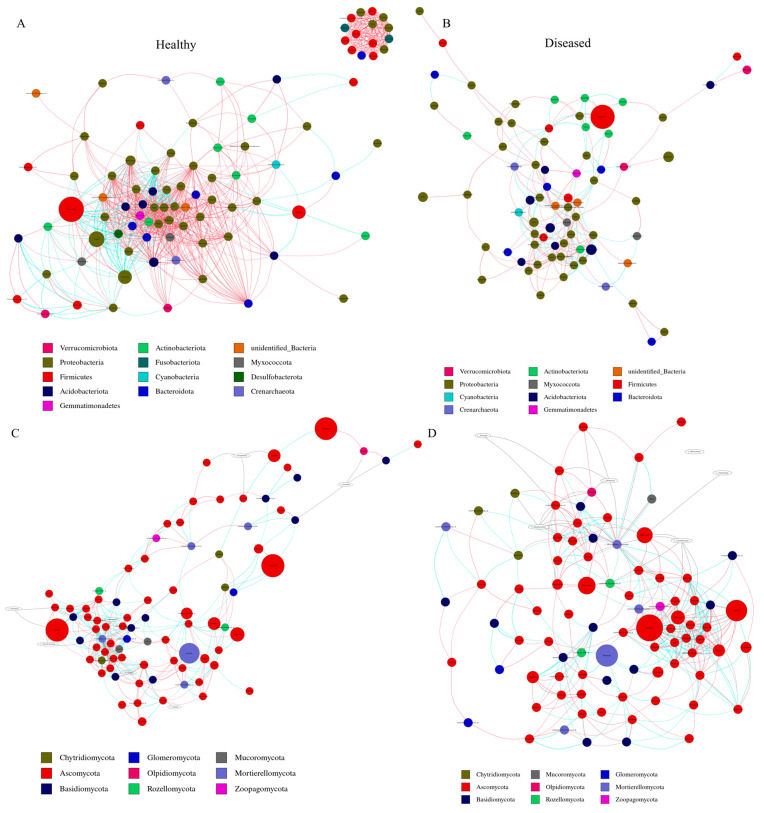
Co-occurrence networks of bacterial and fungal amplicon sequence variants in both healthy and diseased soil. Edges colored in red indicate positive correlations; edges in green indicate negative correlations. (**A**) Bacteria in healthy; (**B**) bacteria in diseased; (**C**) fungi in healthy; and (**D**) fungi in diseased. The different sizes of circles represent the abundance of the taxa of microbes, the larger the circle, the higher the abundance.

The co-occurrence networks for bacteria in healthy (BH) and diseased (BD) states, as well as for fungi in healthy (FH) and diseased (FD) conditions, comprised 96, 81, 95, and 96 nodes, respectively, as illustrated in Figure 9 and detailed in Table S8. A significant proportion of the nodes within the bacterial networks were predominantly classified under the phyla Proteobacteria (BH 44.7%, BD 48.1%) and Firmicutes (BH 18.7%, BD 12.3%). In the fungal networks analyzed, the predominant phyla were Ascomycota (FH 67.3%, FD 66.6%) and Basidiomycota (FH 14.7%, FD 15.6%). The consistent microbial networks for BH, BD, FH, and FD revealed a total of 4560, 3240, 3233, and 4556 co-occurring relationships, respectively. Furthermore, there were 1000, 279, 350, and 429 co-occurring relationships with a correlation coefficient r > 0.7 for each respective network (Figure 10). Additionally, the bacterial community associated with healthy soil displayed a higher proportion of positive interactions (84.70%, as illustrated in Figure 10A) in comparison to that in diseased soil (61.65%, as shown in Figure 10B), implying a reduced level of intergeneric competition in the former. In comparison, a substantial number of positive interactions within the fungal community were recorded in both healthy (51.14%, Figure 10C) and diseased (52.21%, Figure 10D) soil samples. This suggests that the invasion of *F. solani* had a minimal impact on intergeneric competition among fungi.

The complexity and stability of microbial networks in both healthy and diseased soil were assessed using various topological metrics (Table S8). The bacterial network in healthy soil samples exhibited greater complexity, characterized by a higher average degree, clustering coefficient, and connectivity among nodes. Meanwhile, the fungal network in healthy soil samples demonstrated the longest average path length and a higher modularity index. This finding indicates that healthy plants exhibited a greater level of connectivity among adjacent nodes within the microbial network. Furthermore, it suggests that a more cohesive modular structure is likely to emerge from the clustering of these nodes, potentially attributable to the presence of *Trichoderma* or *Bacillus*. Furthermore, exposure of *L. chuanxiong* to the pathogenic fungus *F. solani* resulted in an increase in the diversity and complexity of the fungal network, thereby enhancing the stability of the microbial ecosystem. The findings indicate that healthy soil exhibited more intricate bacterial networks, whereas fungal networks demonstrated increased diversity and complexity in response to pathogen invasion. Collectively, these findings suggest that the invasion of *F. solani* modifies the microbial network, exerting differential impacts on bacterial and fungal populations.

## 4. Discussion

### 4.1. Effects of Root Rot on Yields of Ligusticum chuanxiong Plants

*Ligusticum chuanxiong*, a widely recognized medicinal herb with global significance, is characterized by substantial import and export activity. It is noted for its diverse therapeutic properties, particularly in the realms of cardiovascular protection and hepatoprotection [22]. The *L. chuanxiong* rhizome is extensively utilized in clinical practice owing to its diverse therapeutic properties, which encompass antioxidant effects, hepatoprotective capabilities, and the promotion of blood circulation [23,24]. Consequently, the yield of the rhizome serves as a significant indicator of the growth of *L. chuanxiong*. Root rot, a prevalent soilborne disease caused by *F. solani*, has a substantial adverse effect on both the yield and quality of *L. chuanxiong*, presenting a considerable challenge in its cultivation [3]. Numerous studies have demonstrated that root rot adversely impacts plant growth. Furthermore, as the severity of the disease escalates, the entire root system of the plants exhibits browning and decay, ultimately leading to wilting or even mortality. In a prior investigation, the researchers observed that the fresh weight of *L. chuanxiong* could decrease by a range of 4.25% to 17.56% in the absence of certain biological control agents addressing root rot. The current study reveals that both the fresh weight and dry weight of the shoots and rhizome exhibited a marked decline when *L. chuanxiong* plants were exposed to pathogen infection, with a reduction rate spanning from 47.89% to 70.75%. This suggests that the incidence of root rot has increasingly contributed to a deterioration in the quality of *L. chuanxiong* in recent years. Furthermore, this disease has resulted in varying degrees of quality degradation in several traditional Chinese medicinal herbs, including *Astragalus membranaceus* [25], *Coptis chinensis* [26], *Angelica sinensis* [27], and *Panax notoginseng* [28]. This degradation poses significant challenges to the production of traditional Chinese medicines and incurs substantial economic losses. Consequently, it is imperative to prioritize the management and prevention of root rot.

### 4.2. Response of Alpha Diversity to Root Rot

Microbial communities can be characterized by alpha diversity, which can be quantified using various indices. In particular, the alpha diversity index serves as an indicator of the diversity, richness, and evenness present within the infected soil. In the context of microbial diversity, numerous scholars contend that a high microbial diversity index is indicative of healthy soil conditions and has the potential to mitigate plant diseases [29]. The findings of this study revealed markedly greater bacterial diversity in diseased soil samples. However, no significant differences were noted in the Chao1 and ACE indices, suggesting that bacterial richness and evenness were comparable between healthy and diseased soil.

Concurrently, the quantity of unique bacterial and fungal OTUs that are exclusive to the infected rhizosphere soil was found to be elevated in comparison to that of healthy rhizosphere soil. This finding contradicts the observed alterations in bacterial diversity following infections by prevalent soil-borne diseases [30,31]. However, a similar phenomenon has been documented in the rhizosphere soil of ginseng affected by rusty root disease and in *Hevea brasiliensis* afflicted by red root rot [32]. This phenomenon may be attributed to the occurrence of root rot, which altered the ecological conditions within the rhizosphere soil of *L. chuanxiong*. This alteration likely facilitated the growth and proliferation of certain microorganisms, thereby enhancing the diversity of bacterial species present.

In the context of fungal communities, the alpha diversity observed in diseased soil was found to exceed that of healthy soil, indicating a statistically significant difference in the metrics of observed species, Chao1, and ACE. Prior research has demonstrated a negative correlation between soil health and both the quantity and diversity of fungi, suggesting that certain fungal species can adversely affect plant growth by disrupting root systems, which in turn impacts plant vitality. Research conducted by Siegieda et al. [33] and Zhang et al. [34] indicates that the alpha diversity index of the fungal community in infected rhizosphere soil is greater than that observed in healthy soil. At the same time, it was observed that the diversity and richness of microbial communities in the anthracnose-infected rhizosphere soil were greater than those in healthy soil [35], which aligns with the findings of the present study. However, neither of these investigations provided an explanation for the underlying causes of this phenomenon. According to prior research, plants have the capacity to recruit a variety of rhizosphere microorganisms to fight against soil-borne pathogens upon infection [36,37]. This phenomenon can occasionally lead to an enhancement in microbial diversity. To the best of our understanding, further investigation is required to clarify the precise mechanisms involved. Microbial diversity may not serve as a reliable indicator for assessing whether soil exhibits disease-suppressive or disease-conducive properties, particularly in agricultural soils that are significantly impacted by human activities [38].

### 4.3. Response of Microbial Composition and Structure to Root Rot

The intricate interplay among soil microbial communities is essential for maintaining soil quality and functionality and significantly influences the immunogenic defense mechanisms of host plants [39]. The microbial community within the rhizosphere is recognized as a vital component for supplying essential nutrients, enhancing agricultural productivity, combating soil-borne pathogens, providing protection against both biotic and abiotic stressors, and ensuring the functionality and long-term sustainability of soil ecosystems [10,40]. Therefore, alterations in soil microbiota can serve as indicators of plant growth conditions and serve as a crucial metric for gauging soil health. High-throughput sequencing has uncovered significant differences in microbial species composition between diseased and healthy *L. chuanxiong* plant rhizosphere soil.

The phyla Firmicutes, Proteobacteria, Unclassified, and Acidobacteriota were identified as the most prevalent among both healthy and diseased soils. This finding aligns with earlier research conducted on soils cultivated with American ginseng [41], maize [42], and various other crops [43]. Furthermore, the relative abundance of Firmicutes in healthy soil was significantly greater than that observed in diseased soil. Additionally, *Lactobacillus* and *Bacillus* demonstrated the greatest relative abundance in healthy soil, significantly surpassing the levels observed in diseased soil. Conversely, the relative abundances of genera including *Sphingomonas*, *Candidatus Solibacter*, *MD1*, *RB41*, and *Bryobacter* were found to be increased in diseased soil. The findings of this study align with prior research indicating a significant positive correlation between the relative abundance of *Bacillus* and the survival rates and disease resistance of ginseng [44]. Additionally, an increase in the relative abundance of *Sphingomonas* was observed in cucumbers infected with *Ralstonia solanacearum* [8] and in Chinese cabbage infected with *Plasmodiophora brassicae* [45]. *Lactobacillus* [46], *Bacillus* [47], and *Sphingomonas* [48] species are classified within the phyla Firmicutes and Proteobacteria, respectively. These microorganisms have been documented to significantly contribute to abiotic stress tolerance, pathogen suppression, and plant growth promotion. Based on our findings, it is plausible to hypothesize that *Lactobacillus*, *Bacillus*, and *Sphingomonas* species may serve as biocontrol agents against *L. chuanxiong* root rot; however, further investigation is warranted to substantiate this assertion.

In relation to fungi, our research identified Ascomycota, Mortierellomycota, and Basidiomycota as the predominant phyla. This finding aligns with previous studies conducted on soils cultivated with ginseng [44], American ginseng [38], and strawberries [34]. In addition, Ascomycota and Mortierellomycota showed higher abundance levels in healthy soil compared to diseased soil, whereas the abundance of Chytridiomycota and Basidiomycota was found to be lower in healthy soil. The observed trend regarding Ascomycota contradicts the findings of Yang et al. [49], who documented a higher abundance of Ascomycota in powdery mildew-infected strawberry roots compared to noninfected strawberry plants. This discrepancy is further supported by the results of Gu et al. [50], which indicated an increased abundance of Ascomycota in diseased cucumbers affected by *Fusarium* wilt. In contrast, the trend observed in Basidiomycota aligns with the findings of Zheng et al. [51], who reported a reduction in the relative abundance of Basidiomycota following root rot infection. The observed discrepancy among these studies may be attributed to the significant variability of the Ascomycota taxon across different soil types. This suggests that microorganisms classified at the phylum level are not reliable biomarkers for assessing the potential of a given soil to promote disease. Nonetheless, the findings indicate that the fluctuations in the relative abundance of Ascomycota and Basidiomycota are significantly associated with the root rot of *L. chuanxiong*. However, it is essential to further investigate whether the relationship between Basidiomycetes and Ascomycota is driven by nutritional factors, niche competition, or antagonistic interactions. It is noteworthy that, at the genus level, the relative abundance of *Trichoderma* in healthy soil was approximately five times greater than that observed in infected soil. This genus is known to suppress pathogens and may serve as a potential biocontrol agent, as documented in earlier studies [52]. In contrast, the relative abundance of *Fusarium* exhibited a 36.60% increase in infected soil. The heightened relative abundance of *Fusarium* may serve as a critical factor contributing to the onset of root rot in *L. chuanxiong*. *Fusarium* species, known to induce root rot, represent a significant threat to a wide range of crops worldwide. Extensive research has established that *Fusarium* is a principal pathogen affecting *L. chuanxiong* cultivation. Root rot is anticipated to manifest during the entire growth cycle of *L. chuanxiong* [12].

Furthermore, we conducted an analysis of the correlation between the disease index of root rot and the concentrations of *Trichoderma* and *Bacillus*. Our findings indicated that the hyphae growth of *F. solani* on PDA medium was markedly suppressed by the incorporation of the fungi *Trichoderma* and the bacteria *Bacillus*. Furthermore, a notable negative correlation was identified between the disease index and the concentrations of *Trichoderma* and *Bacillus*. This finding aligns with earlier research conducted on *Fusarium oxysporum*-infected red kidney beans [53] and *Plasmodiophora brassicae*-infected Chinese cabbage [30].

### 4.4. Relationship Between Multiple Soil Environmental Factors and Microbial Communities

Environmental factors, including temperature, humidity, light, and the physicochemical properties of soil, significantly influence the interactions among plants, pathogens, and biocontrol microorganisms. These interactions subsequently lead to physiological changes in host plants and impact the progression of diseases. Alterations in soil characteristics resulting from environmental changes have a direct impact on plant performance and the corresponding microbial communities [54]. Previous research has indicated that soil AP is a significant factor influencing the dynamics of plant microbiome assembly [55]. In our research, RDA analysis confirmed that pH, OM, AK, and TP were critical factors influencing the composition of both bacterial and fungal communities. Additionally, CEC and the concentration of soil TN were found to have a significant impact on the assembly of fungal communities. The findings align with prior research indicating that soil pH, OM, TN, and CEC are the primary environmental variables influencing the assembly of plant microbiomes [45]. Furthermore, the Mantel test indicated that there was no significant correlation between the rhizosphere bacteria and the soil environmental factors. In contrast, a significant positive correlation was found between the pH levels and the concentration of soil OM with the rhizosphere fungi. Moreover, *Trichoderma* exhibited a notable positive correlation with the levels of AK while demonstrating a negative relationship with the severity of root rot. A prior investigation indicated that certain species of *Trichoderma* swiftly colonized a restricted niche within the rhizosphere of soil affected by root rot, thereby mitigating the damage caused by the disease [56]. This finding aligns with the results of the current study. Furthermore, the concentration of soil OM demonstrated a significant influence on *Gemmatimonas*, aligning with prior research findings that indicate plants have the ability to recruit microbes that are resistant to environmental stress, thereby influencing the composition of their rhizosphere microbial communities [57].

### 4.5. Microbial Co-Occurrence Networks

The factors influencing alterations in the rhizosphere microbial community are multifaceted and varied, encompassing elements such as plant species [58] and nutrient availability [59]. Consequently, the specific mechanisms that regulate the assembly of the plant microbiome, contingent upon the growth environments of the plants and their management, are highly intricate and, to a certain degree, unpredictable. In order to determine a causal relationship between shifts in the rhizosphere microbial community and disease index, it is imperative that additional experiments, as outlined in the research conducted by Zhou et al. [60], be undertaken in subsequent studies [38]. In particular, it is essential to isolate the potentially beneficial microbes that have been enriched and to evaluate their disease-suppressing effects in vivo.

The complex interactions among microorganisms play a vital role in the functioning of micro-ecosystems within soil environments. Co-occurrence network analysis provided novel insights into the complex microbial communities and the intricate interactions within microbial communities. We constructed and analyzed the associations between microorganisms in rhizosphere soil following infection with *F. solani* using this method. Our findings indicated that the bacterial network within healthy rhizosphere soil exhibited greater complexity and a higher frequency of positive interactions, implying that the bacterial community in healthy soil demonstrated a tendency towards cooperation rather than intergeneric competition. The findings support the perspective that co-occurrence networks characterized by high connectivity are more conducive to plant growth, thereby improving their resilience to environmental disturbances [61]. The fungal correlations present in *F. solani*-affected soils exhibited greater complexity and diversity compared to those found in healthy soils. This observation is consistent with the findings reported by Guo et al. [53]. Likewise, a similar phenomenon was documented in an additional study conducted by Tan et al. [62]. Consequently, we hypothesize that the increased diversity of associations among microorganisms may be attributed to competition for nutrient resources or ecological niches. Alternatively, the heightened antagonistic interactions observed between fungi and pathogens may result from the disruption of microbial equilibrium caused by pathogen invasion [63,64]. Furthermore, the bacterial network in healthy individuals exhibited greater complexity, characterized by the highest average clustering coefficient and degree of connectivity among nodes. Conversely, the fungal network in healthy individuals demonstrated the highest average path length and modularity index. This finding aligns with the perspective that extended average path lengths are indicative of sluggish responses and heightened resistance of microorganisms to disturbances induced by environmental changes [53]. Furthermore, modular community structures are advantageous for the maintenance of community stability, thereby facilitating the regulation of pathogen proliferation and colonization. Moreover, given that network stability is closely linked to modularity, the elevated modularity observed in fungal communities within a healthy rhizosphere contributes to enhanced network stability [65]. This study further corroborated this finding, indicating that the bacterial community in the rhizosphere exhibited greater sensitivity to root rot compared to the fungal community. Our research indicates that root rot resulted in a reduction of complexity within bacterial communities while simultaneously enhancing the complexity of fungal communities. Additionally, it was observed that root rot exerted a lesser impact on intergeneric competition among fungi. Therefore, it can be inferred that bacterial communities exhibited a greater susceptibleness to root rot. These findings highlight that the rhizosphere microbial community associated with healthy *L. chuanxiong* plants demonstrates a notable level of structural and functional stability. However, it is important to note that diversity and richness alone do not guarantee the stability of the microbial community’s structure and function. However, the microbial community in the rhizosphere of healthy plants shows enhanced resilience and stability in the face of pathogenic disturbances.

In a word, harnessing the rhizosphere microbial communities through rhizosphere microbiome engineering is considered a promising strategy to increase crop production, reduce disease susceptibility, and enhance crop stress tolerance; therefore, it is able to reduce agrochemical inputs. As such, it is of great significance to analyze how plants regulate rhizosphere microecology to control the occurrence of diseases, which is beneficial to explore targeted, applicable, concise and efficient environmental-friendly biological control methods from rhizosphere microorganisms, disease-inhibiting substances and functional metabolism. Also there is an urgent need to explore various effective microbiome-engineering approaches to establish beneficial and stable plant−microbe interactions to improve crop health and agroecosystem sustainability.

## 5. Conclusions

This research investigates the impact of *L. chuanxiong* root rot on plant yields, the composition and structure of the rhizosphere microbial community, soil physicochemical properties, microbial co-occurrence networks, and the interrelationships among these factors. The findings indicate that root rot has a substantial negative impact on the yields of *L. chuanxiong* rhizoma, and it significantly reduced the yield of *L. chuanxiong*, especially the rhizoma. It was observed that root rot led to an increase in the number of unique OTUs of both bacterial and fungal communities within the rhizosphere soil. Furthermore, it resulted in alterations to the alpha diversity and composition of the microbial community, with a notable enrichment of pathogenic fungi, such as *Fusarium*, and the recruitment of beneficial bacteria, including *Gemmatimonas*. It can be seen that the microbial composition and structure in the rhizosphere of *L. chuanxiong* showed complex and subtle changes after root rot infection. Some beneficial microorganisms, such as Firmicutes, Ascomycota, and Mortierellomycota, or *Bacillus* and *Trichoderma*, were recruited for resisting the invasion of pathogens. Additionally, root rot contributed to the reconfiguration of the microbial co-occurrence network, characterized by a reduction in the complexity of bacterial communities while simultaneously enhancing the complexity of fungal communities. In addition, soil pH and OM were found to be critical factors in shaping microbial community structures, which in turn have a substantial impact on the severity of root rot. Moreover, the concentrations of *Trichoderma* and *Bacillus* exhibited significant negative correlations with root rot severity. These findings suggest that these microorganisms could be developed and utilized as potential biocontrol agents against root rot, contingent upon further assessment of their colonization capabilities and biocontrol efficacy in field conditions. The findings offer a theoretical framework for elucidating the microecological mechanisms underlying *L. chuanxiong* root rot as well as for developing ecological strategies for its prevention and control, approached from the standpoint of microbial ecology.

## Figures and Tables

**Table 1:** Correlation between fresh weight, dry weight, and the root rot disease index.

Category	Regression Equation	R^2^
Fresh weight of shoot	y = −10.31x + 74.85	0.985
Fresh weight of rhizoma	y = −27.67x + 175	0.956
Dry weight of shoot	y = −2.134x + 15.01	0.805
Dry weight of rhizoma	y = −11.46x + 67.35	0.954

## Data Availability

The original contributions presented in the study are included in the article/supplementary material; further inquiries can be directed to the corresponding authors.

## Supplementary Materials

The following supporting information can be downloaded at: https://www.mdpi.com/article/10.3390/microorganisms12112350/s1, Figure S1: The hyphae growth of the pathogen *Fusarium solani* on PDA medium 5 days after inoculation. (A) Application of *Fusarium solani* alone. (B) Confrontational application of *Fusarium solani* and *Trichoderma*; Figure S2: The hyphae growth of the pathogen *Fusarium solani* on PDA medium 7 days after inoculation. (A) Application of *Fusarium solani* alone. (B) Confrontational application of *Fusarium solani* and *Bacillus*; Figure S3: The hyphae growth of the pathogen *Fusarium solani* on PDA medium 5 days after inoculation. (A) Application of *Fusarium solani* alone. (B) Combined application of *Fusarium solani* in conjunction with non-volatile organic compounds derived from *Trichoderma*; Table S1: Number of reads remaining before and after QC processes; Table S2: Diversity and richness of the *Ligusticum chuanxiong* rhizosphere soil microbial communities at the genus level between the healthy and diseased plants; Table S3: Relativate abundance of bacteria at phylum level based on gene prediction of co-assembled contigs (%); Table S4: Relativate abundance of fungi at phylum level based on gene prediction of co-assembled contigs (%); Table S5: Relativate abundance of bacteria at genus level based on gene prediction of co-assembled contigs (%); Table S6: Relativate abundance of fungi at genus level based on gene prediction of co-assembled contigs (%); Table S7: The detail information for soil environmental factors; Table S8: Topological properties of the rhizosphere microbiome-associated networks of healthy and root rot-diseased plants of *Ligusticum chuanxiong*.

## References

[1] Huang M., Fu Y., He J., Jia X., Wang Y., Qiu Y.L., Zhao J., Xu P., Zeng Y., Gao P. (2024). Effects of electron beam irradiation on microbial load and physicochemical qualities of *Ligusticum chuanxiong* Hort. Radiat. Phys. Chem..

[2] Song S.M., Chen A.Q., Zhu J.Q., Yan Z.C., An Q.J., Zhou J.Y., Liao H., Yu Y.M. (2021). Structure basis of the caffeic acid O-methyltransferase from *Ligusiticum chuanxiong* to understand its selective mechanism. Int. J. Biol. Macromol..

[3] Li L.Q., Zheng P.F., Tao X.X., Han G.Q., He D.M. (2024). Study on the antagonistic effect of *Fusarium avenaceum* in *Ligusticum chuanxiong* Hort against root rot disease. Pha. Clinic. Chin. Mat. Med..

[4] Abdelaziz A.M., Hashem A.H., El-Sayyad G.S., El-Wakil D.A., Selim S., Alkhalifah D.H., Attia M.S. (2023). Biocontrol of soil borne diseases by plant growth promoting rhizobacteria. Trop. Plant Pathol..

[5] Li X.G., Chen D.L., Carrión V.J., Daniel R., Yin S., Dong Y.H., Zhang T.L., Wang X.X., Manuel D.B. (2023). Acidification suppresses the natural capacity of soil microbiome to fight pathogenic *Fusarium* infections. Nat. Commun..

[6] Yang S.D., Liu H.W., Xie P.H., Wen T., Shen Q.R., Yuan J. (2023). Emerging pathways for engineering the rhizosphere microbiome for optimal plant health. J. Agric. Food Chem..

[7] Zhang J.H., Wei L.F., Yang J., Ahmed W., Wang Y.T., Fu L.N., Ji G.H. (2020). Probiotic consortia: Reshaping the rhizospheric microbiome and its role in suppressing root-rot disease of panax notoginseng. Front. Microbiol..

[8] Deng X.H., Zhang N., Li Y.C., Zhu C.Z., Qu B.Y., Liu H.J., Li R., Bai Y., Shen Q.R., Joana F.S. (2022). Bio-organic soil amendment promotes the suppression of *Ralstonia solanacearum* by inducing changes in the functionality and composition of rhizosphere bacterial communities. New Phytol..

[9] Zhu F.Y., Fang Y., Wang Z.W., Wang P., Yang K.K., Xiao L.T., Wang R.Z. (2022). Salicylic acid remodeling of the rhizosphere microbiome induces watermelon root resistance against *Fusarium oxysporum* f. sp. *niveum* infection. Front. Microbiol..

[10] Trivedi P., Leach J.E., Tringe S.G., Sa T.M., Singh B.K. (2020). Plant-microbiome interactions: From community assembly to plant health. Nat. Rev. Microbiol..

[11] Zhou X., Wang J.T., Liu F., Liang J.M., Zhao P., Tsui C.K.M., Cai L. (2022). Cross-kingdom synthetic microbiota supports tomato suppression of *Fusarium* wilt disease. Nat. Commun..

[12] Li J.S. (2016). Research into Root Rot Disease of *Ligusticum chuanxiong* Hort.: Investigation of the Incidence and Identification of the Fungal Pathogens. Master’s Thesis.

[13] (2007). Determination of Soil pH.

[14] Zhao Z.B., He J.Z., Geisen S., Han L.L., Wang J.T., Shen J.P., Wei W.X., Fang Y.T., Li P.P., Zhang L.M. (2019). Protist communities are more sensitive to nitrogen fertilization than other microorganisms in diverse agricultural soils. Microbiome.

[15] Fei Y.F., Huang S.Y., Zhang H.B., Tong Y.Z., Wen D.S., Xia X.Y., Wang H., Luo Y.M., Barceló D. (2020). Response of soil enzyme activities and bacterial communities to the accumulation of microplastics in an acid cropped soil. Sci. Total Environ..

[16] Xie J., Wu Z., Zhang X.Y., Peng T., Yang C.M., Zhang J.J., Liang J. (2021). Diversity and structural characteristics of soil microbial communities in different habitats of wild *Lilium regale* Wilson in Wenchuan area. Bioengineered.

[17] Zhang X., Liu S., Wang J., Huang Y., Freedman Z., Fu S., Liu K., Wang H., Li X., Yao M. (2020). Local community assembly mechanisms shape soil bacterial Β diversity patterns along a latitudinal gradient. Nat. Commun..

[18] Feng X., Wang Q.L., Sun Y.H., Zhang S.W., Wang F.Y. (2022). Microplastics change soil properties, heavy metal availability and bacterial community in a Pb-Zn-contaminated soil. J. Hazard. Mater..

[19] Song X.D., Liu F., Wu H.Y., Cao Q., Zhong C., Yang J.L., Li D.C., Zhao Y.G., Zhang G.L. (2020). Effects of long-term K fertilization on soil available potassium in East China. Catena.

[20] Faust K., Raes J. (2016). CoNet app: Inference of biological association networks using Cytoscape [version 2; referees: 2 approved]. F1000Research.

[21] Banerjee S., Walder F., Büchi L., Meyer M., Held A.Y., Gattinger A., Keller T., Charles R., van der Heijden M.G.A. (2019). Agricultural intensifcation reduces microbial network complexity and the abundance of keystone taxa in roots. ISME J..

[22] Chen Z.J., Zhang C., Gao F., Fu Q., Fu C.M., He Y., Zhang J.M. (2018). A systematic review on the rhizome of *Ligusticum chuanxiong* Hort. (*Chuanxiong*). Food Chem. Toxicol..

[23] Chinese Pharmacopeia Committee (2020). Pharmacopoeia of People’s Republic of China.

[24] Shi J.H., Li R.Y., Yang S.Y., Phang Y., Zheng C.W., Zhang H.M. (2020). The protective effects and potential mechanisms of *Ligusticum chuanxiong*: Focus on anti-inflammatory, antioxidant, and antiapoptotic activities. Evid-Based. Compl. Alt..

[25] Zhang A.M., Li X.R., Guo B.M., Chen X. (2021). Screening, identification, and biocontrol effect of antagonistic bacteria against *Astragalus membranaceus* root rot. Acta Agric. Boreali-Occident. Sin..

[26] Song X.H., Mei P.Y., Dou T., Liu Q.D., Li L.Y. (2023). Multi-omics analysis reveals the resistance mechanism and the pathogens causing root rot of *Coptis chinensis*. Microbiol. Spectr..

[27] Mu R.R., Liu Y., Lan Q.Q., Zhou Q., Wang X.T., Wang Y.L., Su X., Tian Y.Q. (2024). Characterizing the pathogenicity and mycotoxin production capacity of *Fusarium* spp. causing root rot of *Angelica sinensis* in China. Plant Dis..

[28] Wang H.L., Hai J., Qu Y., Cui X.M., Liu D.Q., Liu G.Z. (2024). Function and regulation of a chitinase gene during *Panax notoginseng* defense response to root rot. J. Appl. Res. Med. Aroma..

[29] Vukicevich E., Lowery T., Bowen P., Úrbez-Torres J.R., Hart M. (2016). Cover crops to increase soil microbial diversity and mitigate decline in perennial agriculture. A Review. Agron. Sustain. Dev..

[30] Ni H.P., Zong R., Sun J.J., Wu Y.X., Yu L., Liu Y.Y., Liu J., Ju R.C., Sun X.L., Zheng Y.L. (2022). Response of bacterial community to the occurrence of clubroot disease in Chinese cabbage. Front. Micbiol..

[31] He Y.H., Huang Y.H., Lu R.M., Yang R.S., Wei Y.N., Liang W.H. (2024). Responses of avocado rhizosphere soil microbial community and its co-occurrence network to root rot disease. J. Cent. South Univ. Forest. Technol..

[32] Tu M., Cai H.B., Peng Y.L., Guan X., Fu X., Zeng X., Hu Y.S. (2021). Structures and biodiversity of microbial communities in rhizosphere soil of red root rot disease and healthy *Hevea brasiliensis*. Chin. J. Trop. Crop..

[33] Siegieda D., Panek J., Frąc M. (2023). Plant and soil health in organic strawberry farms-greater importance of fungal trophic modes and networks than α-diversity of the mycobiome. Appl. Soil Ecol..

[34] Zhang M.L., Kong Z.R., Fu H.J., Shu X.L., Xue Q.H., Lai H.X., Guo Q. (2023). Rhizosphere microbial ecological characteristics of strawberry root rot. Front. Microbiol..

[35] Su D.F., Chen S.Y., Zhou W.X., Yang J.Y., Luo Z.W., Zhang Z.R., Tian Y.X., Dong Q.E., Shen X.M., Wei S.J. (2022). Comparative analysis of the microbial community structures between healthy and anthracnoseinfected strawberry rhizosphere soils using Illumina sequencing technology in Yunnan Province, Southwest of China. Front. Microbiol..

[36] Xiong W., Li R., Ren Y., Liu C., Zhao Q.Y., Wu H.S., Jousset A., Shen Q.R. (2017). Distinct roles for soil fungal and bacterial communities associated with the suppression of vanilla *Fusarium* wilt disease. Soil. Biol. Biochem..

[37] Huang W.J., Sun D.L., Fu J.T., Zhao H.H., Wang R.H., An Y.X. (2020). Effects of continuous sugar beet cropping on rhizospheric microbial communities. Genes.

[38] Bi Y.M., Zhang X.M., Jiao X.L., Li J.F., Peng N., Tian G.L., Wang Y., Gao W.W. (2023). The relationship between shifts in the rhizosphere microbial community and root rot disease in a continuous cropping American ginseng system. Front. Microbiol..

[39] Stringlis I.A., Yu K., Feussner K., de Jonge R., Van Bentum S., Van Verk M.C., Berendsen R.L., Bakker P.A.H.M., Feussner I., Pieterse C.M.J. (2018). MYB72-dependent coumarin exudation shapes root microbiome assembly to promote plant health. Proc. Natl. Acad. Sci. USA.

[40] Carrión V.J., PerezJaramillo J., Cordovez V., Tracanna V., de Hollander M., RuizBuck D., Mendes L.W., van Ijcken W.F.J., GomezExposito R., Elsayed S.S. (2019). Pathogen-induced activation of disease-suppressive functions in the endophytic root microbiome. Science.

[41] Jiang J.L., Yu M., Hou R.P., Li L., Ren X.M., Jiao C.J., Yang L.J., Xu H. (2019). Changes in the soil microbial community are associated with the occurrence of *Panax quinquefolius* L. root rot diseases. Plant Soil..

[42] Li D.W., Yang Y.H., Zhao Y.L., Tian G.L., Li M.S., Qiu H.S., Zhou X.G. (2022). Differing roles of bacterial and fungal communities in cotton fields by growth stage. Agronomy.

[43] Shen Z.Z., Xue C., Penton C.R., Thomashow L.S., Zhang N., Wang B.B., Ruan Y.Z., Li R., Shen Q.R. (2019). Suppression of banana Panama disease induced by soil microbiome reconstruction through an integrated agricultural strategy. Soil. Biol Biochem..

[44] Li X.Y., Liu Q., Gao Y.G., Zang P., Zheng T. (2024). Effects of a co-bacterial agent on the growth, disease control, and quality of ginseng based on rhizosphere microbial diversity. BMC Plant Biol..

[45] Liu Y., Lai J., Sun X.F., Huang L., Sheng Y.Z., Zhang Q.F., Zeng H.L., Zhang Y.C., Ye P.S., Wei S.G. (2024). Comparative metagenomic analysis reveals rhizosphere microbiome assembly and functional adaptation changes caused by clubroot disease in Chinese cabbage. Microorganisms.

[46] Núria D., Gemma R., Jordi C., Irene D., Jesús F., Esther B., Francesco S., Emilio M., Anna B. (2019). Biological control of bacterial plant diseases with *Lactobacillus plantarum* strains selected for their broad-spectrum activity. Ann. Appl. Biol..

[47] Wang J., Wang J.R., Liu T.T., Li X., Gao J., Jiang Y., Chen C.Q. (2023). *Bacillus amyloliquefaciens* FG14 as a potential biocontrol strain against rusty root rot of *Panax ginseng*, and its impact on the rhizosphere microbial community. Biol. Control.

[48] Mazoyon C., Hirel B., Pecourt A., Catterou M., Gutierrez L., Sarazin V., Dubois F., Duclercq J. (2023). *Sphingomonas sediminicola* is an endosymbiotic bacterium able to induce the formation of root nodules in pea (*Pisum sativum* L.) and to enhance plant biomass production. Microorganisms.

[49] Yang J.Y., Wei S.J., Su D.F., Zhang Z.R., Chen S.Y., Luo Z.W., Shen X.M., Lai Y.H., Jamil A., Tong J.Y. (2020). Comparison of the rhizosphere soil microbial community structure and diversity between powdery mildew-infected and noninfected strawberry plants in a greenhouse by high-throughput sequencing technology. Curr. Microbiol..

[50] Gu Z.C., Wang M., Wang Y., Zhu L.X., Mur L.A.J., Hu J., Guo S.W. (2020). Nitrate stabilizes the rhizosphere fungal community to suppress fusarium wilt disease in cucumber. Mol. Plant Microbe Interact..

[51] Zheng Y.X., Wang J.M., Zhao W.L., Cai X.J., Xu Y.L., Chen X.L., Yang M., Huang F.Y., Yu L., He Y.S. (2022). Effect of bacterial wilt on fungal community composition in rhizosphere soil of tobaccos in tropical Yunnan. Plant Pathol. J..

[52] Poletto T., Fantinel V.S., Muniz M.F.B., Quevedo A.C., Strahl M.A., Poletto I., Stefenon V.M. (2024). Efficacy of five *Trichoderma* species against anthracnose pathogens in pecan through mycoparasitism and antibiosis. J. Crop Health.

[53] Guo Z.F., Zhang J.X., Liu Z.B., Li Y., Li M., Meng Q.X., Yang Z.P., Luo Y., Zhang Q., Yan M. (2024). *Trichoderma harzianum* prevents red kidney bean root rot by increasing plant antioxidant enzyme activity and regulating the rhizosphere microbial community. Front. Microbiol..

[54] Zhang S.N., Wang Y., Sun L.T., Qiu C., Ding Y.Q., Gu H.L., Wang L.J., Wang Z.S., Ding Z.T. (2020). Organic mulching positively regulates the soil microbial communities and ecosystem functions in tea plantation. BMC Microbiol..

[55] Wu W.X., Huang X.Q., Zhang L., Yang X.X., Li H.Z., Liu Y. (2020). Crucifer clubroot disease changes the microbial community structure of rhizosphere soil. Acta Ecol. Sin..

[56] Gao P.X., Qi K., Han Y.J., Ma L.G., Zhang B., Zhang Y.L., Guan X.M., Qi J.S. (2023). Effect of *Trichoderma viride* on rhizosphere microbial communities and biocontrol of soybean root rot. Front. Microbiol..

[57] Gao M., Xiong C., Gao C., Tsui C.K.M., Wang M.M., Zhou X., Zhang A.M., Cai L. (2021). Disease-induced changes in plant microbiome assembly and functional adaptation. Microbiome.

[58] Liu Y.Z., Liu Y., Zeng C.L., Wang J.Y., Nyimbo W.J., Jiao Y.Y., Wu L.K., Chen T., Fang C.X., Lin W.X. (2022). Intercropping with *Achyranthes bidentata* alleviates *Rehmannia glutinosa* consecutive monoculture problem by reestablishing rhizosphere microenvironment. Front. Plant Sci..

[59] Rosinger C., Keiblinger K.M., Rousk J., Sandén H. (2022). Shifts in microbial stoichiometry upon nutrient addition do not capture growth-limiting nutrients for soil microorganisms in two subtropical soils. Biogeochemistry.

[60] Zhou X.G., Zhang X.H., Ma C.L., Wu F.Z., Jin X., Andreote F.D., Wei Z. (2022). Biochar amendment reduces cadmium uptake by stimulating cadmium-resistant PGPR in tomato rhizosphere. Chemosphere.

[61] Faust K., Raes J. (2012). Microbial interactions: From networks to models. Nat. Rev. Microbiol..

[62] Tan L., Zeng W.A., Xiao Y.S., Li P.F., Gu S.S., Wu S.L., Zhai Z.G., Feng K., Deng Y., Hu Q.L. (2021). 2021. Fungi-bacteria associations in wilt diseased rhizosphere and endosphere by interdomain ecological network analysis. Front. Microbiol..

[63] García-Bayona L., Comstock L.E. (2018). Bacterial antagonism in host associated microbial communities. Science.

[64] Hu J., Wei Z., Kowalchuk G.A., Xu Y.C., Shen Q.R., Jousset A. (2020). Rhizosphere microbiome functional diversity and pathogen invasion resistance build up during plant development. Environ. Microbiol..

[65] Jacopo G., Tim R., Stefano A. (2016). Modularity and stability in ecological communities. Nat. Commun..

