# Supplementary material for: Response of Yields, Soil Physiochemical Characteristics, and the Rhizosphere Microbiome to the Occurrence of Root Rot Caused by Fusarium solani in Ligusticum chuanxiong Hort
Source: Microorganisms. 2024 Nov 18;12(11):2350. doi: 10.3390/microorganisms12112350 (PMC11596405; doi:10.3390/microorganisms12112350)
Supplement: Supplementary file 1 [file microorganisms-12-02350-s001.zip › Supplementary Figures.pdf]

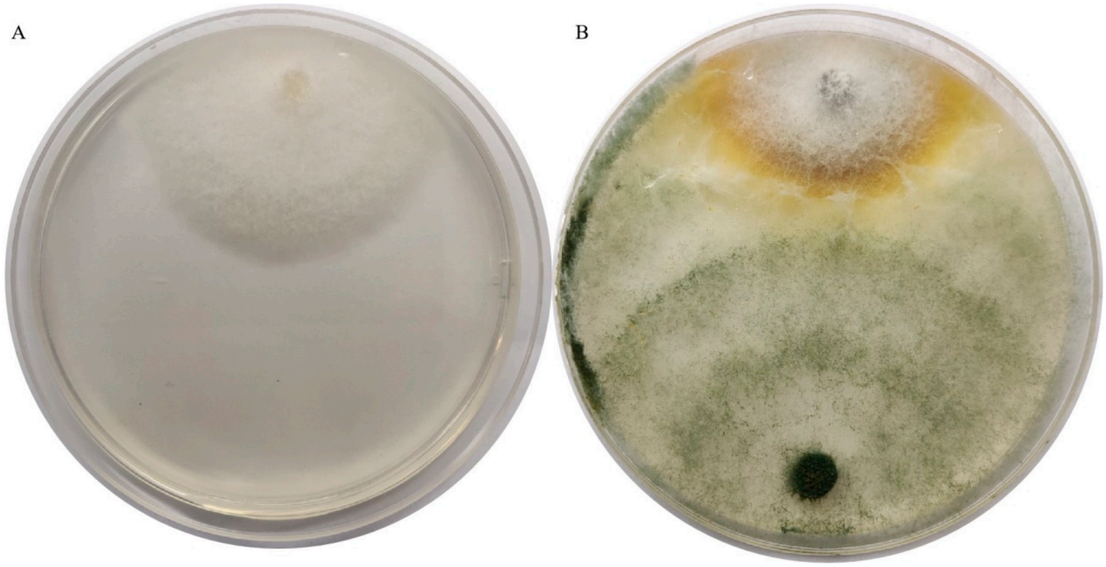

Figure S1: The hyphae growth of the pathogen *Fusarium solani* on PDA medium 5 days after inoculation. (A) Application of *Fusarium solani* alone. (B) Confrontational application of *Fusarium solani* and *Trichoderma*.

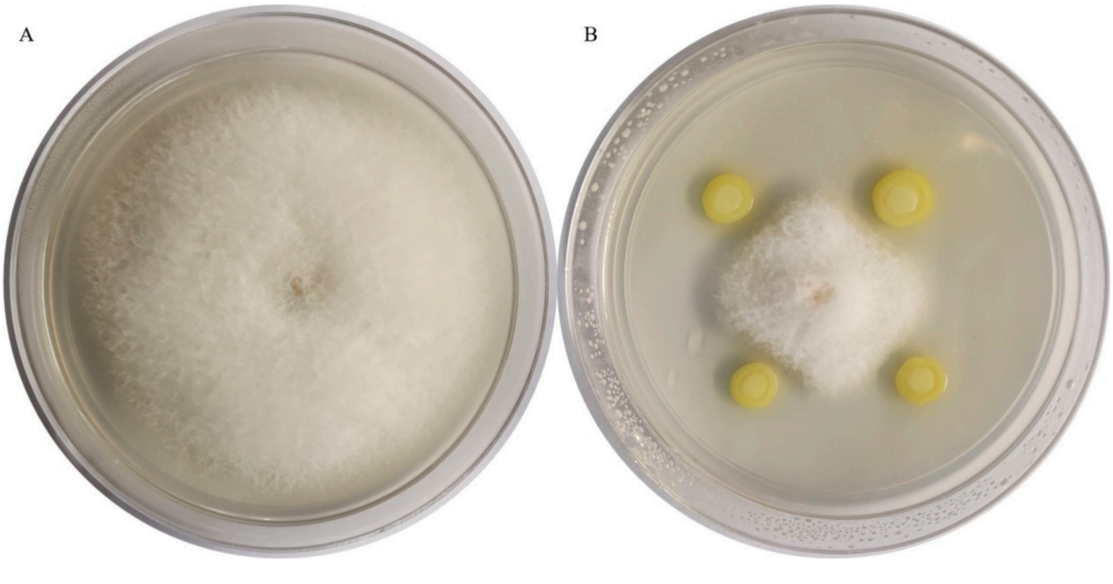

Figure S2: The hyphae growth of the pathogen *Fusarium solani* on PDA medium 7 days after inoculation.(A) Application of *Fusarium solani* alone. (B) Confrontational application of *Fusarium solani* and *Bacillus.*

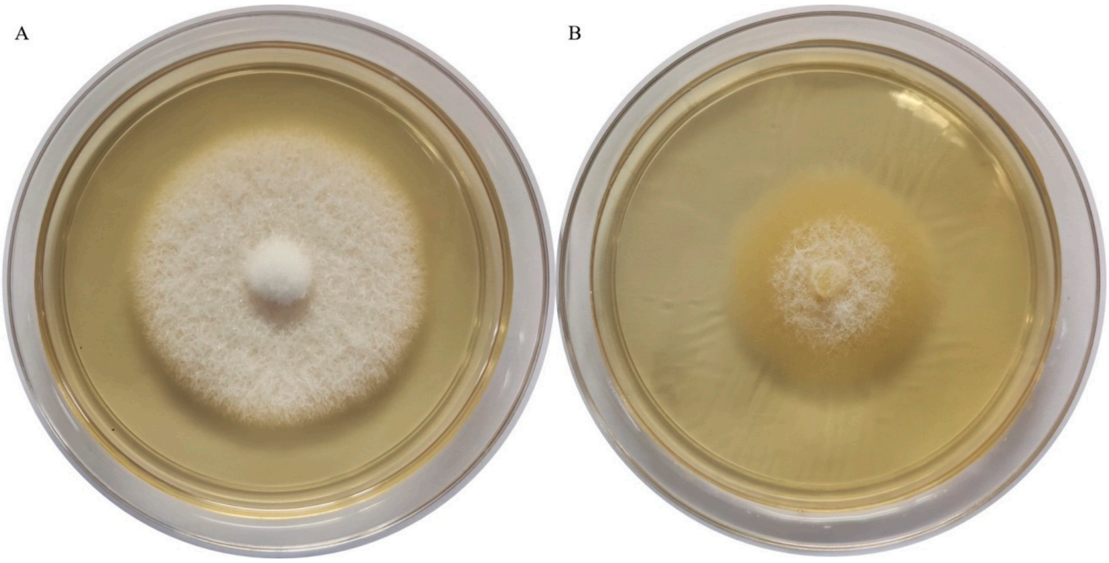

Figure S3: The hyphae growth of the pathogen *Fusarium solani* on PDA medium 5 days after inoculation. (A) Application of *Fusarium solani* alone. (B) Combined application of *Fusarium solani* in conjunction with non-volatile organic compounds derived from *Trichoderma.*
